# Health Workforce Staffing in Immigration Prisons

**DOI:** 10.1007/s11606-025-09837-4

**Published:** 2025-10-24

**Authors:** Joseph Nwadiuko, Erik Carrillo, Annette M. Dekker, Amy Zeidan, A. Katrina Nelson, Diana Simmes, Parveen Parmar

**Affiliations:** 1 Department of Medicine, Perelman School of Medicine, University of Pennsylvania, Philadelphia, USA; 2 Leornard Davis Institute of Health Economics, University of Pennsylvania, Philadelphia, PA USA; 3 Department of Family Medicine, David Geffen School of Medicine at University of California-Los Angeles, Los Angeles, CA USA; 4 Department of Emergency Medicine, David Geffen School of Medicine at University of California-Los Angeles, Los Angeles, CA USA; 5 Department of Emergency Medicine, Emory School of Medicine, Atlanta, GA USA; 6 Department of Health Policy and Management, Yale School of Public Health, New Haven, CA USA; 7 Behind Bars Data Project, UCLA School of Law, Los Angeles, CA USA; 8 Department of Emergency Medicine, Harbor-UCLA Medical Center, David Geffen School of Medicine at University of California-Los Angeles, Los Angeles, CA USA

## INTRODUCTION

There are ongoing concerns about the healthcare quality in US Immigration and Customs Enforcement (ICE) facilities, with 78% of deaths being related to violations of ICE’s own healthcare standards^1^. Although no prior research has investigated the impact of workforce challenges in ICE, a study evaluating healthcare staffing in US jails demonstrated that qualified providers were rarely on-site, requiring nurses and officers to manage “healthcare responsibilities that exceed their training”^2^. It is unclear whether ICE facilities struggle with similar challenges. This study aims to evaluate ICE facility workforce staffing levels between fiscal years (FY) 2020–2022.

## METHODS

Workforce data was retrieved from 20 facilities for which the federal government (rather than a private contractor) oversaw care^3^. We also tabulated privately contracted workforce data for the Denver Contract Detention Facility (DDCF) from Congressional audit reports^3^. Together, these facilities represent 15% of facilities and 35% of the average daily population of detainees between 2020 and 2022. We calculated the ratio of providers (physicians and advanced practice providers (APP)) to book-ins (i.e., facility entries), the ratio of psychiatrist providers to book-ins, the ratio of providers to clinic visits, and the ratio of psychiatry providers to patients under close supervision for mental health reasons. Clinical utilization and outcome data is drawn from ICE Facility Significant Incident Reports from 2019–2022^3^. Identifying information for a subset of medical providers at government-run facilities was obtained through a Freedom of Information Act Request (FOIA)^3^. We analyzed their credential characteristics for the presence of sanctions. The study was considered exempt by the University of Pennsylvania Institutional Review Board.

## RESULTS

We gathered workforce data on 21 facilities and workforce and population data across 54 facility years. Twelve facilities (57%) lacked continuous physician coverage for at least one year. Ten (48%) facilities had no continuously employed psychiatry providers for at least one year, including five (24%) with no behavioral health providers of any type, two (10%) that reported solitary confinement for mental health reasons in the same year, and six (29%) with attempted suicides in the same year.

Among facility-years with relevant providers, the median ratio of book-ins to continuously available medical providers was, 1527:1 (range: 158:1–6,535:1); book-ins to psychiatrists was 6,152:1 (range 905:1–22,446:1); annual clinic visits to provider was 708:1 (range 52:1–7,214:1) and psychiatry providers to mental health patients under close observation was 25 (range 0–98).

FOIA records revealed 3,239 providers, 1,189 of whom were listed as active and 232 for whom we received identifiable provider-level DEA numbers. We identified 61 physicians, 8 dentists, 62 advanced practice providers, and 101 inactive DEA numbers. 7 physicians (11.7%) had state sanctions against them; for poor medical documentation (1), prescribing with an expired license (1), practicing under the influence or being otherwise unfit (2), and fabricating medical records (1); details on two sanctions were not publicly available.

## DISCUSSION

This study demonstrates that some facilities operate with inadequate staffing. Assuming a typical workflow of 4 patients per hour, at least one facility (South Texas ICE Processing Center) in 2021 might have had more clinical visits (7,214) than providers could have feasibly seen (6,325). These shortages are consistent with an Inspector General Report that found vacancy rates up to 35% for health positions, due in part to the length of background checks (with some applicants failing them), geographical isolation of facilities, and discomfort of practicing within detention settings^4^. This is despite increasing healthcare expenditures from $336.1 million to $373.5 million between FY 2020–2022^5^. Furthermore, over 10% of identifiable physicians had sanctions. By comparison, 0.14% of all physicians receive a first-time sanction each year^6^; it would take 77 years for a hypothetical US physician population to have a similar rate, assuming no workforce turnover.

Study limitations include inability to assess whether individuals are seen by providers with the appropriate level of training, a challenge noted in jails. This also assumes that any given worker is fully assigned to a single facility and not shared across several. This analysis also does not fully account for detention facilities with privately contracted healthcare. However, as the current administration plans to increase deportations, thereby increasing individuals detained, efforts must be made to improve staffing and avoid the use of detention.

## Data Availability

Data can be made available upon request to the authors.
